# Preeclampsia has an association with both platelet count and mean platelet volume: A systematic review and meta-analysis

**DOI:** 10.1371/journal.pone.0274398

**Published:** 2022-09-14

**Authors:** Muluken Walle, Yemataw Gelaw, Fasil Getu, Fikir Asrie, Zegeye Getaneh

**Affiliations:** 1 Medicallaboratory Science Department, College of Medicine and Health Sciences, Jigjiga University, Jigjiga, Ethiopia; 2 Department of Hematology and Immunohematology, School of Biomedical and Laboratory Sciences, College of Medicine and Health Sciences, University of Gondar, Gondar, Ethiopia; Royal College of Surgeons in Ireland, IRELAND

## Abstract

**Background:**

Preeclampsia (PE) is a pregnancy-specific disorder characterized by endothelial dysfunction, and activation of the coagulation system. Alteration of PLT parameters is the common hematological abnormality observed in women with PE. The main aim of this study was to systematically review previous studies from around the world to generate evidence about the relationship between platelet count (PC) and PE, as well as mean platelet volume (MPV) and PE, by calculating the pooled weighted mean difference (WMD) of PC and MPV between PE and normotensive (NT) groups.

**Methods:**

Relevant articles which were published in the English language from January 10, 2011, to January 10, 2021, were systematically searched through PubMed, Web of Science, and African journals online. In addition, reference probing of published articles searching was employed through Google Scholar and Google for searching grey literature. The methodological qualities of articles were assessed using Joana Brigg’s institute critical appraisal checklist. A random-effects model was used to estimate pooled WMD of PLT parameters between the two groups with the respective 95% confidence intervals (CI) using Stata version 11.0. The I^2^ statistics and Egger’s regression test were used to assess heterogeneity and publication bias among included studies, respectively.

**Results:**

A total of 25 articles were included in this systematic review and meta-analysis. Of which, 23 studies were used in each PC and MPV analysis. The overall pooled WMD of PC and MPV between PE and NT groups were -41.45 × 10^9^/L [95% CI; -51.8, -31.0] and 0.98 fl [95% CI; 0.8, 1.1], respectively. The pooled WMD revealed that PC decreased significantly in the PE group compared to the NT group while MPV increased significantly in the PE group.

**Conclusions:**

This systematic review and meta-analysis indicated that there is a significant decrease in PC and a significant increase in MPV during PE development among pregnant women. As a result, a change in these parameters among pregnant women may indicate the development of PE.

## Introduction

Preeclampsia (PE) is a pregnancy-specific medical disorder that is characterized by activation of the coagulation system, and endothelial cell dysfunction [1]. It is diagnosed by elevated blood pressure (BP), a systolic BP (SBP) of at least 140 mmHg, or a diastolic BP (DBP) of 90 mmHg after 20^th^ GW with proteinuria ≥ 1+ in urine dipstick [2]. Women with PE present diverse signs and symptoms associated with multiple organ systems, including headache, thrombocytopenia, severe hypertension, chest pain, pulmonary edema, low oxygen saturation, and abnormal liver and kidney function [3].This disorder is a major cause of maternal and fetal morbidity and mortality [4]. Around 5% of pregnancies are complicated by PE worldwide [5]. World Health Organization (WHO) estimated that the incidence of PE is higher in developing countries than in developed countries [6].

The placenta has always been a central figure in the etiology of PE [4].A two-stage model was developed to easily explain its complex pathogenesis [7]. First, deficient spiral artery remodeling in the uterus is caused by inadequate fetal trophoblast invasion of uterine tissue. The resulting abnormal implantation reduces maternal blood flow to the placenta and fetus, ultimately leading to the development of placental ischemia and an increase in oxidative stress [8, 9]. The second stage of PE starts when the placenta responds to progressive ischemia or hypoxia; the placenta may secret and release reactive oxygen species, chemokines, pro-inflammatory cytokines, and anti-angiogenic factors into the maternal circulation that contribute to endothelial damage [7, 10, 11].

Among the potential mediators, the balance between pro-angiogenic factors and anti-angiogenic factors is particularly clinically important [12].The disproportionate levels of pro-angiogenic factors like vascular endothelial growth factor (VEGF), placental growth factor (PlGF) and transforming growth factor-β (TGFβ), and anti-angiogenic factors such as soluble endoglin (sEng) and soluble fms-like tyrosine kinase-1 (sFlt-1) are believed to cause generalized maternal endothelial dysfunctions [4]. For instance, the elevated levels of sFlt are thought to bind and reduce the bioavailability of VEGF, impairing their endogenous production of nitric oxide and causing vasoconstriction. The production of nitric oxide is induced by VEGF which neutralizes reactive oxygen species and vasoconstrictor signaling [13].

Furthermore, the release of Chemokines and pro-inflammatory cytokines induces inflammation in the maternal circulation, and the release of reactive oxygen species contributes to further placental oxidative stress and endothelial dysfunction [14].

The contact of platelet (PLT) with the injured endothelium activates the coagulation system which leads to an increase in PLT consumption and production [15]. Activation of the coagulation system with increased PLTs aggregation leads to multisystem dysfunction in PE [16, 17]. The elevated consumption of PLTs due to the abnormal coagulation system and PLT activation leads to thrombocytopenia which can be used as an important sign of PE [15, 18]. Mean platelet volume (MPV) is one of the groups of PLT parameters that is mainly related to PLT morphology and proliferation kinetics [19]. It is a marker of PLT size, function, and activation as the number and size of pseudopodia increases during PLT activation [20]. The increased consumption of PLTs obligated the bone marrow to produce and release young and large PLTs [15, 21] leading to an increase in MPV in PE patients [22, 23]. There were studies conducted on the association of PLT parameters with PE, however, the findings were controversial. For instance, several studies [17, 24, 25] found a significant association between PE and platelet count (PC), as well as PE and MPV; on the other hand, some studies [26–28] found non-significant differences in these parameters between PE and NT groups. Therefore, the primary goal of this systematic review and meta-analysis was to determine the pooled weighted mean difference (WMD) of PC and MPV between PE and normotensive (NT) groups and generate evidence about the association between PC and PE, and MPV and PE.

## Materials and methods

### Study design

This systematic review and meta-analysis was performed based on an updated preferred reporting item for systematic review and meta-Analysis (PRISMA) guideline 2020 [29]. This systematic review and meta-analysis analyzed findings from published articles to evaluate the pooled WMD of some PLT parameters between preeclamptic and NT pregnant women globally.

### Eligibility criteria

Studies that met the following criteria were considered eligible for inclusion in this study.

#### Types of studies

We include all published original studies with cross-sectional, case-control, and cohort study designs. There were language restrictions; studies published only in the English language were included. Moreover, we have included studies that were published from January 10, 2011, to January 10, 2021.

#### Types of participants

We included studies that were carried out on the pregnant woman with a primary clinical diagnosis of PE and also having a control NT group.

#### Study area

Those studies that were conducted all over the world.

#### Types of outcome measures

The primary outcome of the study was determining the WMD of PC and MPV between PE and NT groups. Studies reported the outcome of interest (PC and MPV) and express the results as mean and standard deviation (SD) or median and interquartile range (IQR) for both PE and NT groups were included in this study.

#### Publication condition

Studies that meet the eligibility criteria were included regardless of their publication status (published, unpublished and grey literature, etc.).

After a thorough screening of the abstracts and the full texts of the studies, articles having methodological problems were excluded. Studies that did not report the value of PC and MPV in both groups were excluded. Studies whose entire text was not available for free were also excluded. Furthermore, studies conducted among pregnant women, but who had co-morbidities like; HIV/AIDS, malaria, hypertension, and coagulation disorder were excluded from the study.

### Information source

Data were collected through searching previous literature on electronic databases such as PubMed, Science direct, and African journals online using search terms. In addition, reference probing of published articles searching was employed through Google Scholar and Google for searching grey literature.

### Search strategy

This was done independently by three reviewers (MW, ZG, and YG). A systematic search of published articles was performed on electronic databases using search terms. The search terms were developed following the Medical Subject Headings (Mesh) thesaurus in combination with free text key terms, and they were used in combination using Boolean operators like “OR” or “AND”. The searching terms used in electronic databases were “platelet parameter”, “platelet indices”, “platelet count”, “thrombocytes count”, “mean platelet volume”, “mean platelet size”, “MPV”, and “preeclampsia”. During the literature search, the results were limited by study population, language, availability of full text, and date of publication. Relevant articles which were written in the English language and published from January 10, 2011, to January 10, 2021, were systematically searched.

### Selection process

After studies are identified through extensive searching, the search results were imported into EndNote X9 (Thomson Reuters, New York, USA) to organize and remove duplicated articles. After the removal of the duplicates, two authors (MW and YG) independently and meticulously screened the title and abstract of each of the retrieved articles for eligibility. In case of disagreement, consensus on which articles to screen full-text was reached by discussion. If necessary, the third reviewer (ZG) was consulted to make the final decision. Next, the two reviewers independently screened full-text articles for inclusion. Again, in case of disagreement, the consensus was reached on inclusion or exclusion by a discussion with the third reviewer.

### Data collection process

We designed a data extraction form, which two review authors (MW and FA) used to extract data from eligible studies. Reviewers worked independently to extract study details. A third reviewer (ZG) reviewed data extraction, and resolve conflicts. The mean±SD values of PC and MPV in each group were extracted. The results of these parameters that were expressed as median (IQR) were also extracted and changed to mean and SD using a formula that was recommended by Wan et al. [30]. Additionally, the first author’s name, publication year, study country, study design, the sample size of the PE group, the sample size of the NT group, and PE severity status among PE groups were extracted. Moreover, the standard error (SE) of WMD was calculated to do Egger’s test and funnel plot. Extracted data were compared, with any discrepancies being resolved through discussion. Then, MW entered the extracted data into a Microsoft Excel spreadsheet.

#### Data item

- PC: is a laboratory test to measure the number of PLTs per microliter of blood.
- MPV: is a laboratory test used to measure a PLT volume that reflects the bone marrow activity [31].
- PE severity: PE cases are divided into two categories based on the severity of the condition: mild PE and severe PE. Preeclamptic patients can be considered with mild PE when the BP ranged from 140/90-160/110 mmHg, and proteinuria ≥ 1 on a urine dipstick while severe PE when BP is ≥ 160/110 mmHg with proteinuria >3+ on a urine dipstick and edema and other major symptoms [2]. We extracted this data as mild or severe directly from the included studies. If the studies did not classify them as mild and severe, they were taken as undefined PE.

### Risk of bias assessment

The methodological qualities of included studies were appraised in detail using the Joana Brigg’s institute (JBI) tool [32] by two reviewers (FA and FG). The tool consists of 8 items to assess the internal and external validity for cross-sectional studies, 10 items for case-control studies, and 11 items for cohort studies. Each item was assessed as 0 for “not done”, 1 for “done”, UC for “unclear”, and NA for “not applicable”. Any disagreements during the review process were resolved by consensus and when the disagreements continue after discussion, a third person (ZG) was consulted to finally settle the discrepancy. Finally, the overall qualities of the study were scored according to the number of done items per study quality assessment items. Articles with an average score of 50% and above were included in this meta-analysis study.

### Effect measures

We planned to analyze the pooled WMD of PC and MPV and their 95%confidence interval (CI) between PE and NT groups in pair-wise meta-analyses by extracting the value of these parameters in each group from the report of previous studies.

### Statistical analysis and interpretation

After the relevant data had been extracted from the studies using Microsoft excel format, the authors then analyzed the results by using STATA version 11.0 (STATA Corporation, College Station, TX, USA), and it was performed by MW and FG together. The original studies were summarized and presented by using a table and the forest plot. The random-effects model was used for pooling WMD analysis of PLT parameters between groups with the respective 95% CIs.

Heterogeneity between included studies was determined using Higgin’s I-squared statistics. The I^2^ values of 25%, 50%, and 75% were considered as low, medium, and high heterogeneity, respectively [33]. To explore the potential source of heterogeneity, subgroup analysis was carried out by study area, study design, and PE severity. Sensitivity analysis was performed to evaluate the influence of each study on the pooled measures by omitting one single study in each turn and recalculating the pooled WMD for the remainders. Publication bias was evaluated by inspection of funnel plots and Egger’s test (a statistical analog for funnel plots) [34]. All analyses were performed using Stata 11.0 (StataCorp LP, College Station, TX, USA), and *P* < 0 05 was considered to be statistically significant.

## Results

### Search and study selection

**Fig 1:**
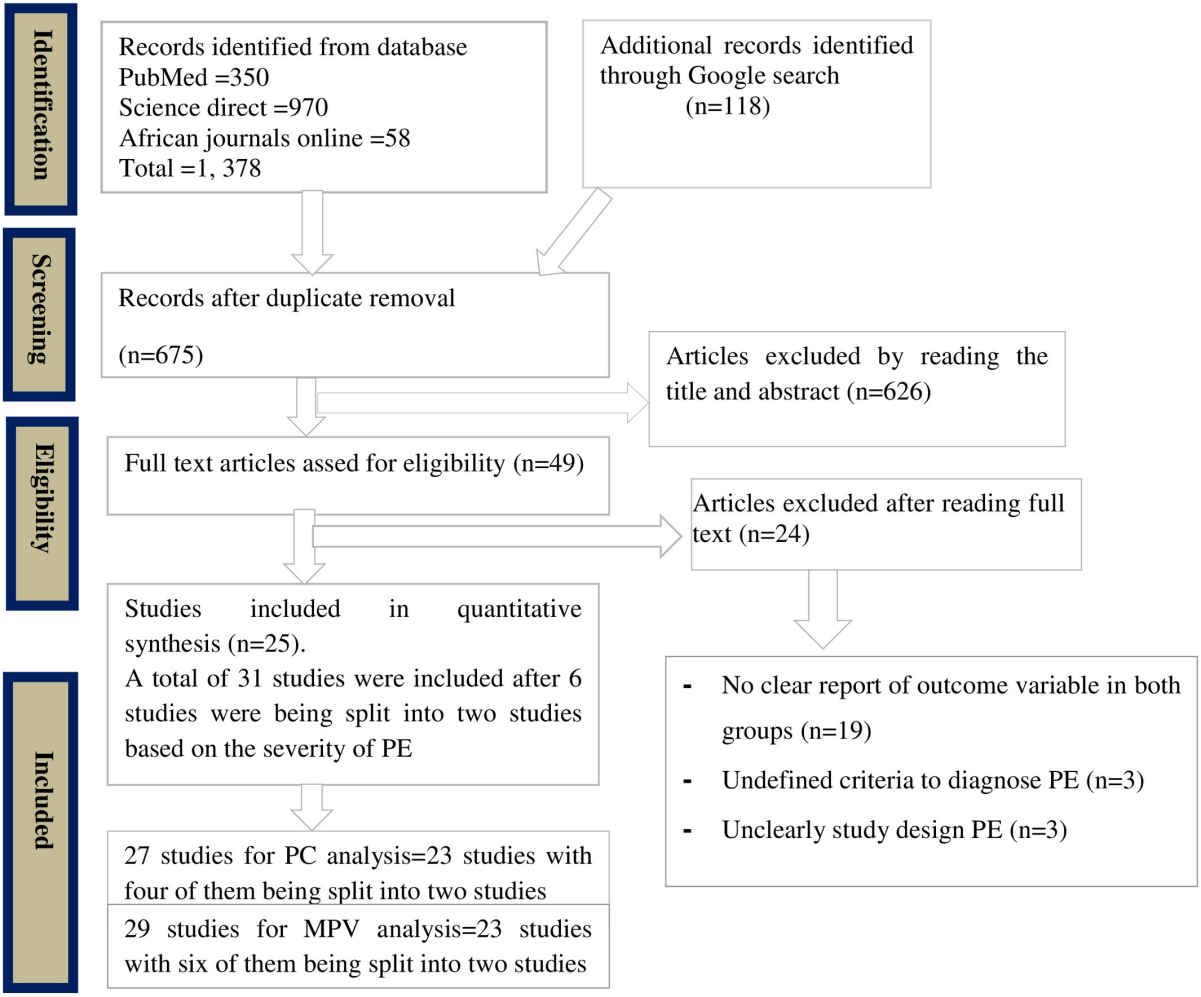
Flow chart to describe the selection of studies for the systematic review and meta-analysis on the association between PC and MPV with PE.

A total of 1, 496 studies were identified through database searching and Google search which were done on pregnant women and published from January 10, 2011, to January 10, 2021, in the English language. After removing duplicates, a total of 675 studies remained for screening. Out of which, 650 articles were excluded after reading their title (626 articles) and full-text (24 articles).Among 24 studies that were excluded after reading the full text, 19of them did not report the outcome variable (PLT parameters) in both groups clearly [1, 10, 18, 22, 35–49], and three studies did not report the criteria to diagnose PE [50–52], and the remaining two studies had not clearly described the study design [53, 54]. Finally, after excluding non-relevant articles, a total of 25 studies that reported the outcome of interest in terms of mean±SD or median (IQR) were included and used for the final analysis (Fig 1).

### Study characteristics

In this systematic review and meta-analysis, a total of 25 articles were included. Out of 25 articles, three were conducted in China [38, 50, 55], four in India [17, 56–58], four in Turkey [16, 26, 27, 59], two in Korea [24, 60], two in Saudi-Arabia [61, 62], two in Egypt [25, 63], two in Sudan [64, 65], two were in Ethiopia [66, 67] and the other four studies were from Bangladesh [68], Brazil [69], Pakistan [70], and Mexico [28]. Of the included studies, 20 of them expressed the result of the outcome with mean±SD and the rest five [24, 26, 58, 59, 61] expressed the results with median (IQR).

A total of 27 studies were included for pooled WMD analysis of PC (Table 1). Of them, eight studies were included by splitting four studies [24, 25, 60, 67] into two based on PE severity. The included studies for PC analysis comprised a total of6, 250 pregnant women (1,943 PE and 4, 307NT). Of preeclamptic women, 319 women were with mild PE, 480 with severe PE and the rest 1,144 had undefined PE.A total of 29 studies were included for the pooled WMD analysis of MPV, with six of them [24, 25, 38, 59, 60, 67] being split into two studies depending on the severity of PE (Table 2).The included studies comprised a total of 6, 609 pregnant women (2,034PE and 4,575NT).

**Table 1:** Summary characteristics of included studies in the pooled WMD estimate of PC between PE and NT groups.

SN	Author	Publication year	Study design	Country	Sample size of cases	Sample size of controls	Mean PC in cases	Mean PC in controls	SD of PC in cases	SD of PC in controls	PE Severity
1	Annam et’al[17]	2011	Case control	India	82	100	155.5	218.4	31.3	28.23	Undefined
2	Freitaset’al[69]	2013	Case control	Brazil	29	28	195.2	234.0	68.8	62.8	Sever
3	Alkholyet’al[25]	2013	Cross sectional	Egypt	50	50	183.94	249.12	37.3	38.35	Mild
4	Alkholyet’al[25]	2013	Cross sectional	Egypt	50	50	139.34	249.12	32.61	38.35	Sever
5	Yang et’al[24]	2014	Prospective cohort	Korea	59	816	234.67	240.67	160.34	158.9	Mild
6	Yang et’al[24]	2014	Prospective cohort	Korea	60	816	207.67	240.67	123.8	158.9	Sever
7	Mazharet’al[70]	2014	Cross sectional	Pakistan	80	60	141.82	210.78	1.70	1.81	Undefined
8	Amitaet’al[57]	2015	Case control	India	50	50	166.9	258.9	71.9	66.9	Undefined
9	Mondal et’al[68]	2015	Cross sectional	Bangladesh	32	32	219.7	300.9	63.7	69.7	Undefined
10	Doğanet’al[16]	2015	Case control	Turkey	119	165	209.4	238.9	90.26	70.79	Undefined
11	Kurt et’al[27]	2015	Cross sectional	Turkey	52	50	281	272	77	75	Undefined
12	AlSheehaet’al[61]	2016	Case control	Saudi-Arabia	60	60	230.25	265.8	77.67	81.27	Undefined
13	Kurtogluet’al[26]	2016	Case control	Turkey	150	100	225.8	227.7	79.0	55.7	Undefined
14	Abasset’al[64]	2016	Case control	Sudan	37	50	236.2	261.34	82.33	62.69	Undefined
15	Mohammed F [65]	2016	Case control	Sudan	60	60	185.5	257.7	78.1	66.6	Undefined
16	Chen et’al[55]	2017	Retrospective cohort	China	125	188	180.72	220.60	58.14	45.56	Undefined
17	Gutierrez et’al[28]	2017	Prospective cohort	Mexico	31	30	181.9	188.2	65.29	47.45	Undefined
18	Kim et’al[60]	2018	Retrospective cohort	Korea	126	471	221.8	237.5	5.6	3.3	Mild
19	Kim et’al[60]	2018	Retrospective cohort	Korea	227	471	194.8	237.5	5.2	3.3	Sever
20	Sitotawet’ al [66]	2018	Cross sectional	Ethiopia	63	63	158.4	194.1	42.7	45.6	Undefined
21	Zhang et’al[50]	2019	Case control	China	100	100	198.54	239.40	64.54	54.53	Undefined
22	Gogoiet’al[56]	2019	Cross sectional	India	67	67	188	200.1	89.7	62.36	Undefined
23	Thaloret’l[58]	2019	Case control	India	30	30	217	241	57.7	54.8	Undefined
24	Elgariet’al[62]	2019	Cross sectional	Saudi-Arabia	80	80	223	228	78.4	80.7	Undefined
25	Hassan et’al[63]	2019	Case control	Egypt	45	40	191.4	229.25	48.16	65.7	Undefined
26	Tesfay et’ al[67]	2019	Cross sectional	Ethiopia	35	140	226	291.6	56.5	58.4	Mild
27	Tesfay et’ al [67]	2019	Cross sectional	Ethiopia	44	140	185.3	291.6	60.2	58.4	Sever

**Table 2:** Summary characteristics of included studies in the pooled WMD estimate of MPV between PE and NT groups.

So	Author	Publication year	Study design	Study place	Sample size of cases	Sample size of controls	Mean MPV of cases	Mean MPV of controls	SD of MPV in cases	SD of MPV in controls	PE Severity
1	Annam et’al[17]	2011	Case control	India	82	100	10.38	8.63	1.65	1.32	Undefined
2	Freitaset’al[69]	2013	Case control	Brazil	29	28	9.6	9.1	1.1	0.9	Sever
3	Alkholyet’al[25]	2013	Cross sectional	Egypt	50	50	9.82	8.50	0.68	0.75	Mild
4	Alkholyet’al[25]	2013	Cross sectional	Egypt	50	50	11.07	8.50	1.08	0.75	Sever
5	Han et’al[38]	2014	Case control	China	53	79	10.6	10.4	1.5	1.6	Mild
6	Han et’al[38]	2014	Case control	China	41	79	11.4	10.4	1.4	1.6	Sever
7	Yang et’al[24]	2014	P. cohort	Korea	59	816	11.07	9.9	3.95	2.5	Mild
8	Yang et’al[24]	2014	P. cohort	Korea	60	816	11.27	9.9	2.7	2.5	Sever
9	Mazharet’al[70]	2014	Cross sectional	Pakistan	80	60	8.56	11.76	1.7	1.2	Undefined
10	Amitaet’al[57]	2015	Case control	India	50	50	9.308	8.89	1.21	0.97	Undefined
11	Mondalet’al[68]	2015	Cross sectional	Bangladesh	32	32	11.55	10.05	0.86	0.71	Undefined
12	Doğanet’al[16]	2015	Case control	Turkey	119	165	9.67	9.11	1.81	1.49	Undefined
13	Kurtogluet’al[26]	2016	Case control	Turkey	150	100	9.7	8.77	6.21	4.66	Undefined
14	Abasset’al[64]	2016	Case control	Sudan	37	50	10.15	9.48	1.10	0.87	Undefined
15	Mohammed F[65]	2016	Case control	Sudan	60	60	10.8	9.8	1.1	1.0	Undefined
16	Chen et’al[55]	2017	R. cohort	China	125	188	10.40	9.49	2.63	1.35	Undefined
17	Gutierrez et’al[28]	2017	P. cohort	Mexico	31	30	11.89	11.5	0.99	1.14	Undefined
18	Yücelet’al[59]	2017	R. cohort	Turkey	27	110	10.34	9.37	7.28	6.87	Mild
19	Yücelet’al[59]	2017	R. cohort	Turkey	82	110	11.12	9.37	7.37	6.87	Sever
20	Kim et’al[60]	2018	R. cohort	Korea	126	471	9.5	8.8	0.1	0.07	Mild
21	Kim et’al[60]	2018	R. cohort	Korea	227	471	9.8	8.8	0.1	0.07	Sever
22	Sitotawet’al[66]	2018	Cross sectional	Ethiopia	63	63	11.28	10.31	0.97	1.13	Undefined
23	Zhang et’al[50]	2019	Case control	China	100	100	11.38	10.17	1.39	1.42	Undefined
24	Gogoiet’al[56]	2019	Cross sectional	India	67	67	9.45	9.02	1.19	1.1	Undefined
25	Thaloret’l[58]	2019	Case control	India	30	30	11.8	10.5	1.25	2.07	Undefined
26	Elgariet’al[62]	2019	Cross sectional	Saudi Arabia	80	80	10.6	10.4	1.2	1.1	Undefined
27	Hassan et’al[63]	2019	Case control	Egypt	45	40	10.86	9.32	1.42	0.95	Undefined
28	Tesfayet’al[67]	2019	Cross sectional	Ethiopia	35	140	11.5	8.4	2.1	0.9	Mild
29	Tesfayet’al[67]	2019	Cross sectional	Ethiopia	44	140	12.3	8.4	1.7	0.9	Severe

### The association of platelet count with preeclampsia

**Fig 2:**
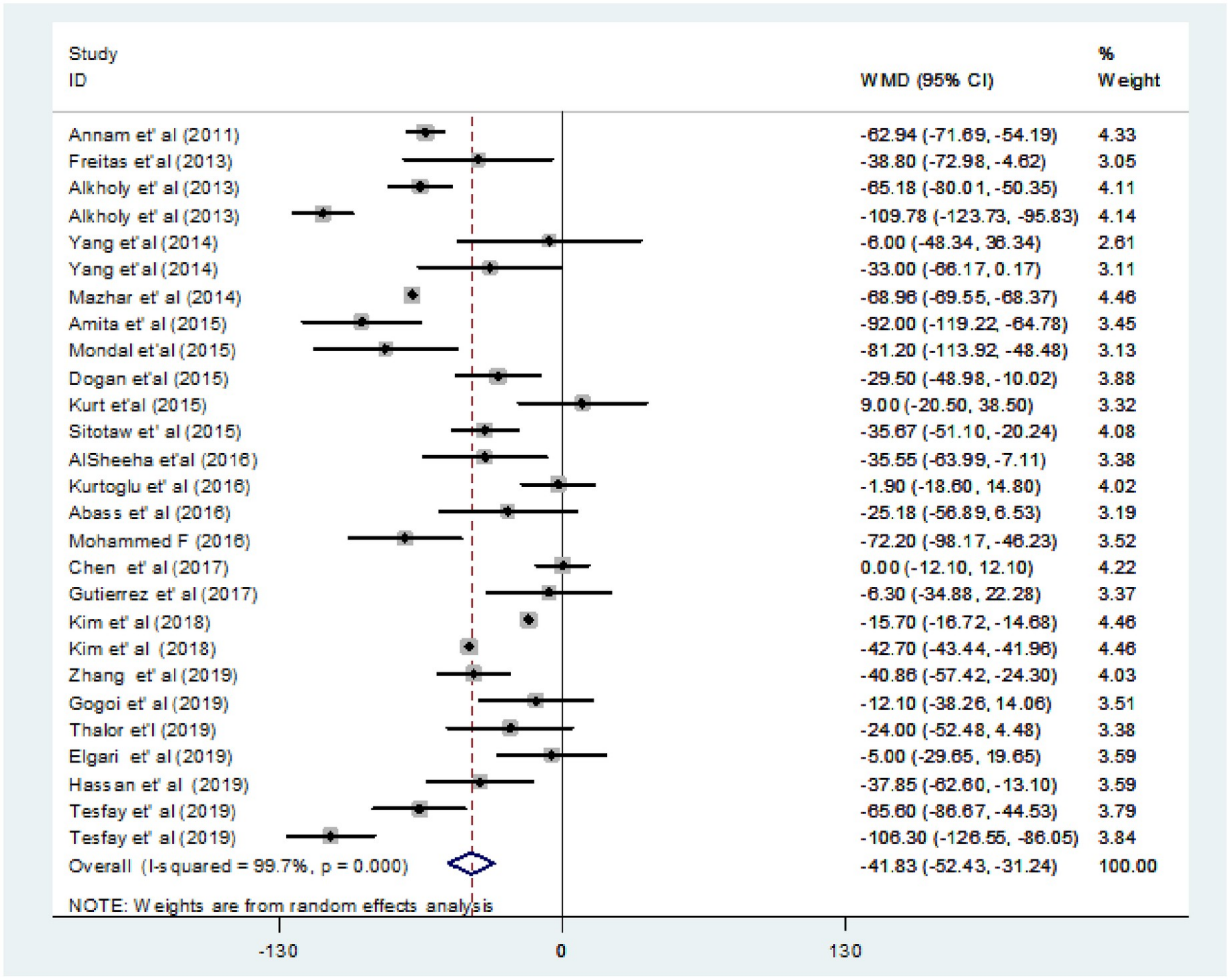
Forest plot of included studies for the pooled WMD estimate of PC between PE and NT groups.

We performed a random-effect meta-analysis of pooled WMD for PC on the extracted 27 studies. The overall pooled WMD revealed that PC decreased significantly in the PE group compared to the NT group [WMD: -41.5×10^9^/L, 95% CI; -51.8×10^9^/L, -31.0×10^9^/L] (Fig 2).The estimated pooled mean of PC in PE and NT groups was 190.1×10^9^/L [95% CI;164.2×10^9^/L, 216.1×10^9^/L] and 232.6×10^9^/L [95% CI;144.2× 10^9^/L, 321.1×10^9^/L], respectively.

### The association of mean platelet volume with preeclampsia

**Fig 3:**
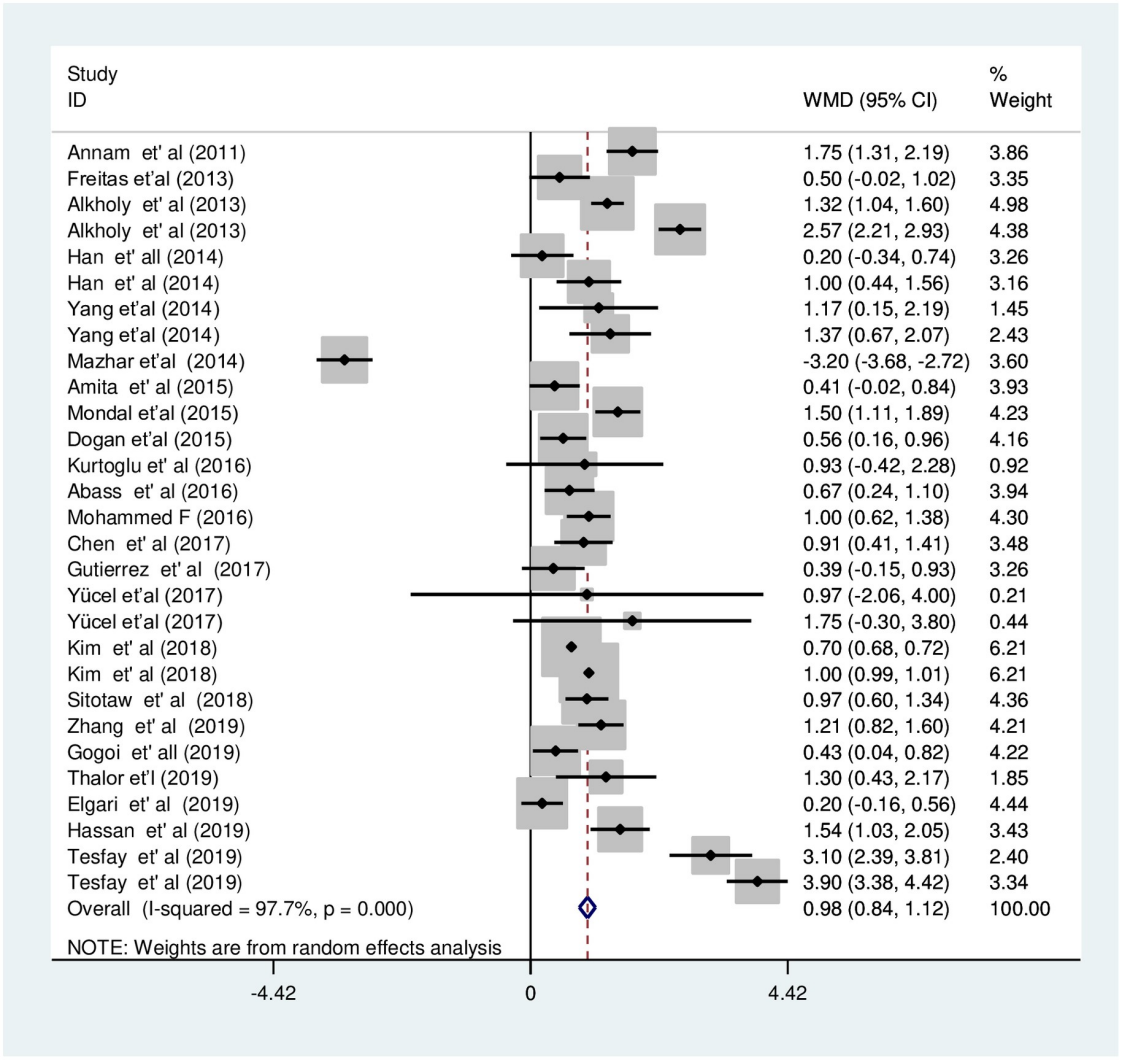
Forest plot of included studies for the pooled WMD estimate of MPV between PE and NT groups using a random effect model.

The random-effects meta-analysis for MPV showed that the estimated pooled mean of MPV in PE patients and NT pregnant women was 9.8fl [95% CI; 9.6fl, 10.1fl] and 8.8fl [95% CI; 8.7fl, 8.9fl], respectively. The overall pooled WMD analysis revealed that MPV values were significantly increased in the PE group when compared with the NT group [WMD: 0.98fl; 95%CI; 0.8, 1.1] (Fig 3).

### Heterogeneity and publication bias analysis

The existence of heterogeneity and publication bias was determined within included studies. The heterogeneity of the included study was high according to Higgin’s I-squared statistics (I^2^ = 99.7%; p<001 for PC and I^2 =^ = 97.7%; p<001 for MPV). The heterogeneity indicated that there was a high variation of studies which lead us to subgroup analysis. The subgroup analyses were performed based on the year of publication, PE severity, and region where the studies were conducted. However, there was no significant reduction in heterogeneity.

**Fig 4:**
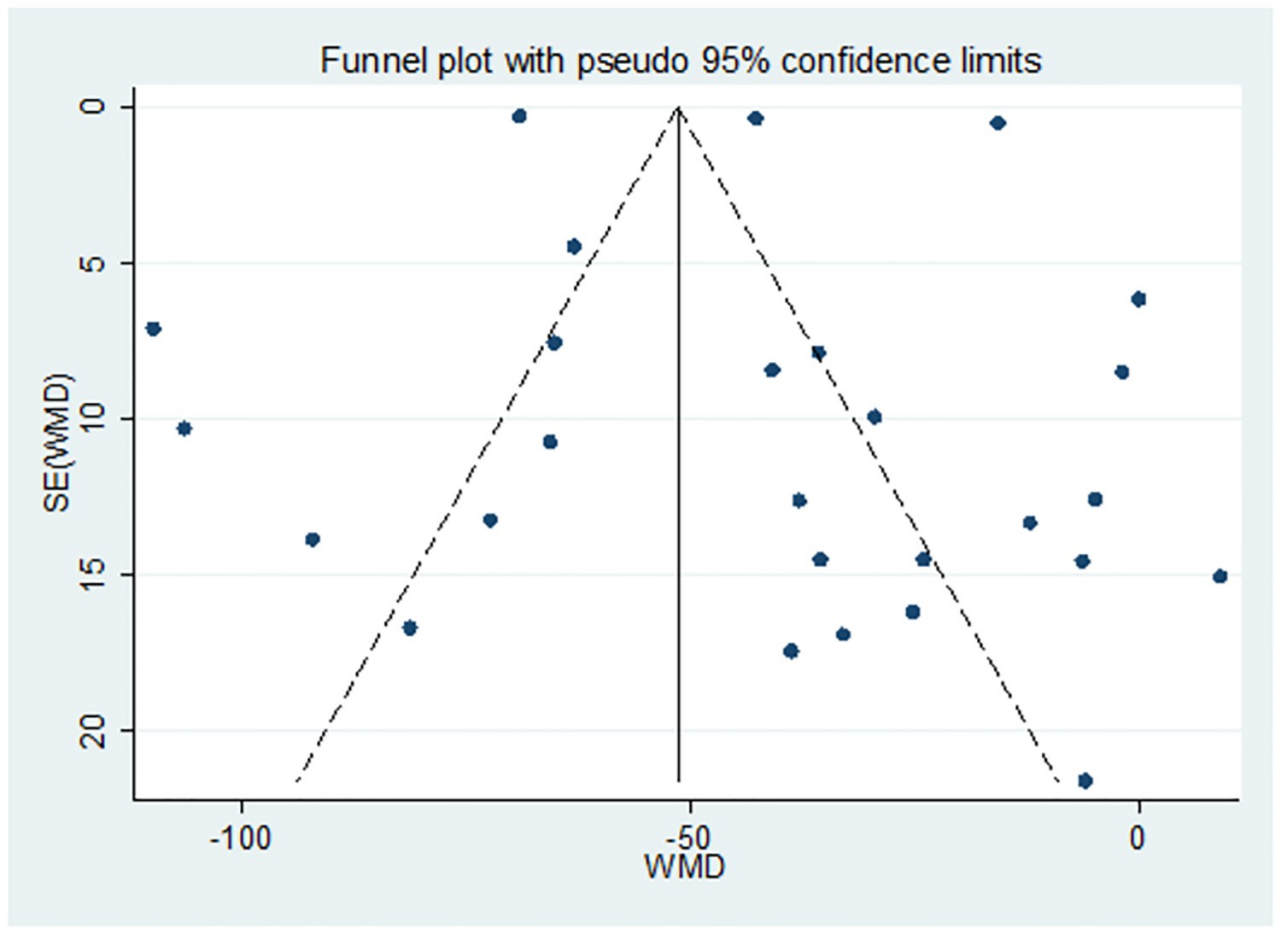
Funnel plot for the pooled WMD estimate of PC between PE and NT groups.

**Fig 5:**
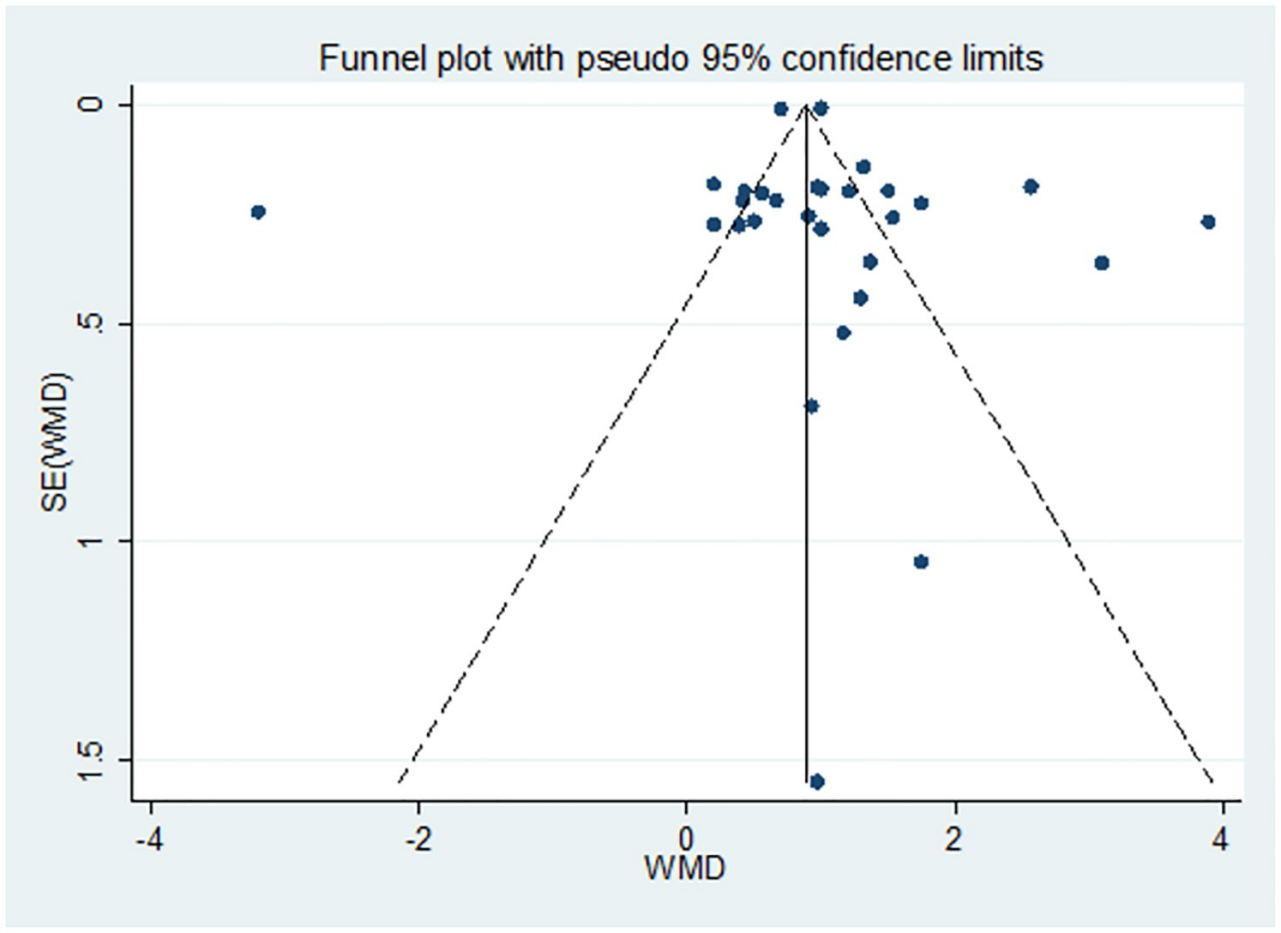
Funnel plot for the pooled WMD estimate of MPV between PE and NT groups.

The Egger’s test was used to determine the presence of publication bias for the analysis of pooled WMD of PLT parameters between PE and NT pregnant women. The P-value in Egger’s test showed that the publication bias was marginally insignificant in all PLT parameter analyses [P = 0.565 for PC and P = 0.811 for MPV]. All the values were greater than 5%; indicating no evidence of publication bias within included studies (Tables 3 & 4) and no need for trim and fill analysis. Besides, a funnel plot was also depicted to illustrate the presence/absence of publication bias in all PC (Fig 4) and MPV (Fig 5) analyses.

**Table 3:** Egger’stest of publication bias for the pooled WMD estimate of PC between PE and NT groups.

Std_Eff	Coef.	Std. Err.	T	P>|t|	[95% Conf. Interval]
Slope	52.44276	4.401098	11.92	0.000	43.37853, 61.50699
Bias	-2.26591	3.97081	-0.57	0.573	-10.44395, 5.912126

**Table 4:** Egger’s test of publication bias for the pooled MD estimate of MPV between PE and NT groups.

Std_Eff	Coef.	Std. Err.	T	P>|t|	[95% Conf. Interval]
Slope	0.8849026	0.0420218	21.06	0.000	0.7986811, 0.9711241
Bias	0.3039611	1.350903	0.23	0.824	-2.467863, 3.075785

### Subgroup analysis

#### Subgroup analysis in estimated pooled weighted mean difference of platelet count between Preeclampsia and normotensive groups

**Fig 6:**
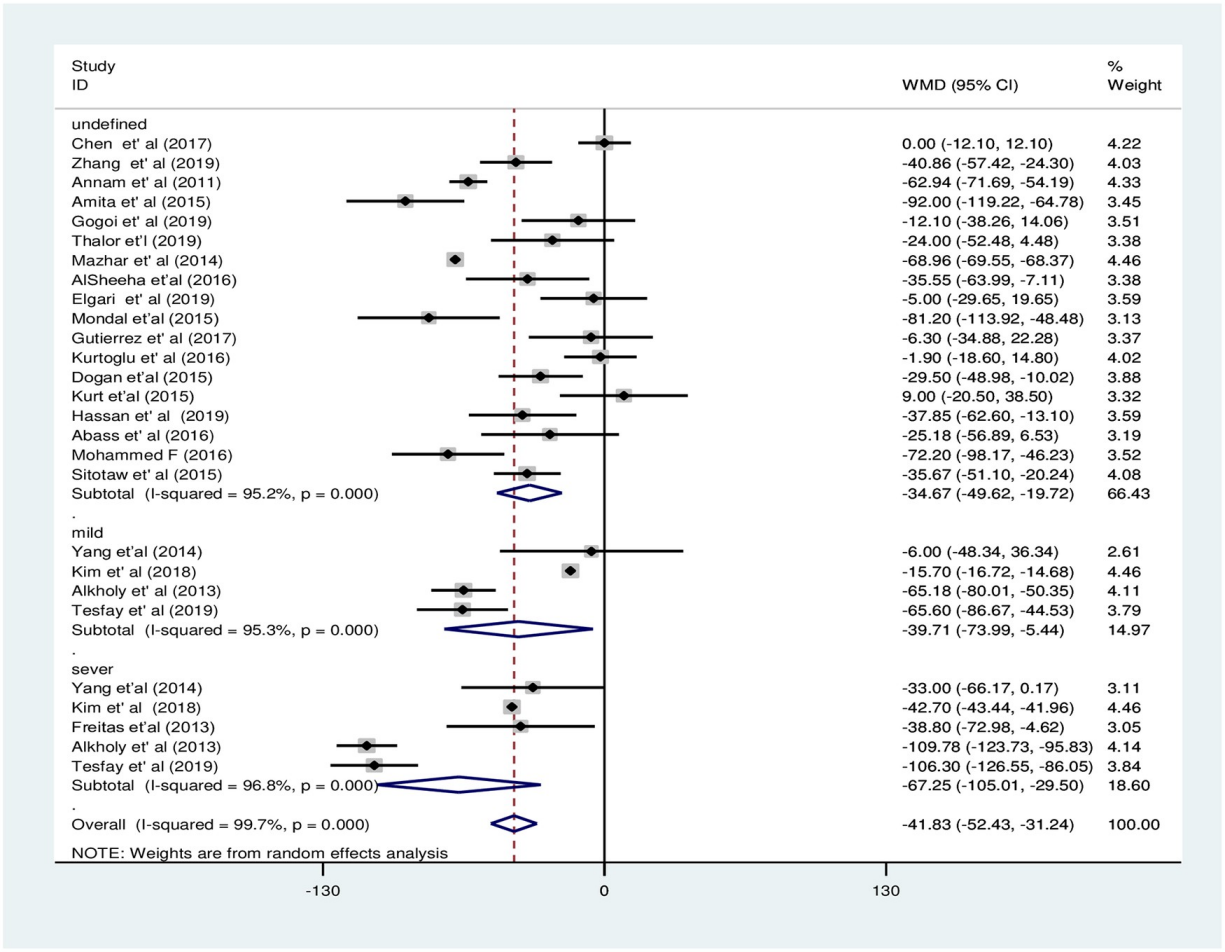
Forest plot of subgroup analysis for the pooled WMD estimate of PC by PE severity.

**Fig 7:**
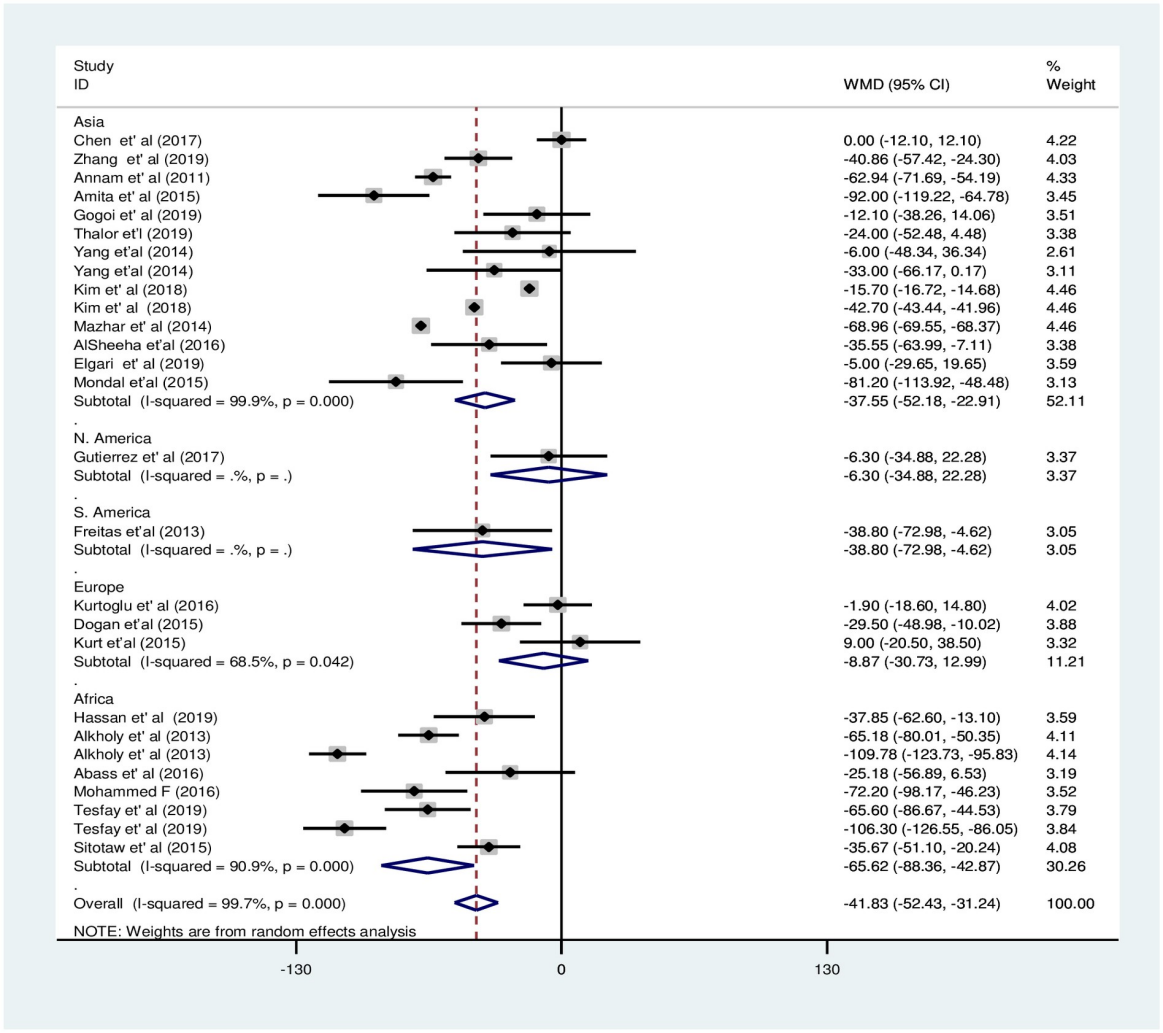
Forest plot of subgroup analysis for the pooled WMD estimate of PC by continent using random effect model.

**Fig 8:**
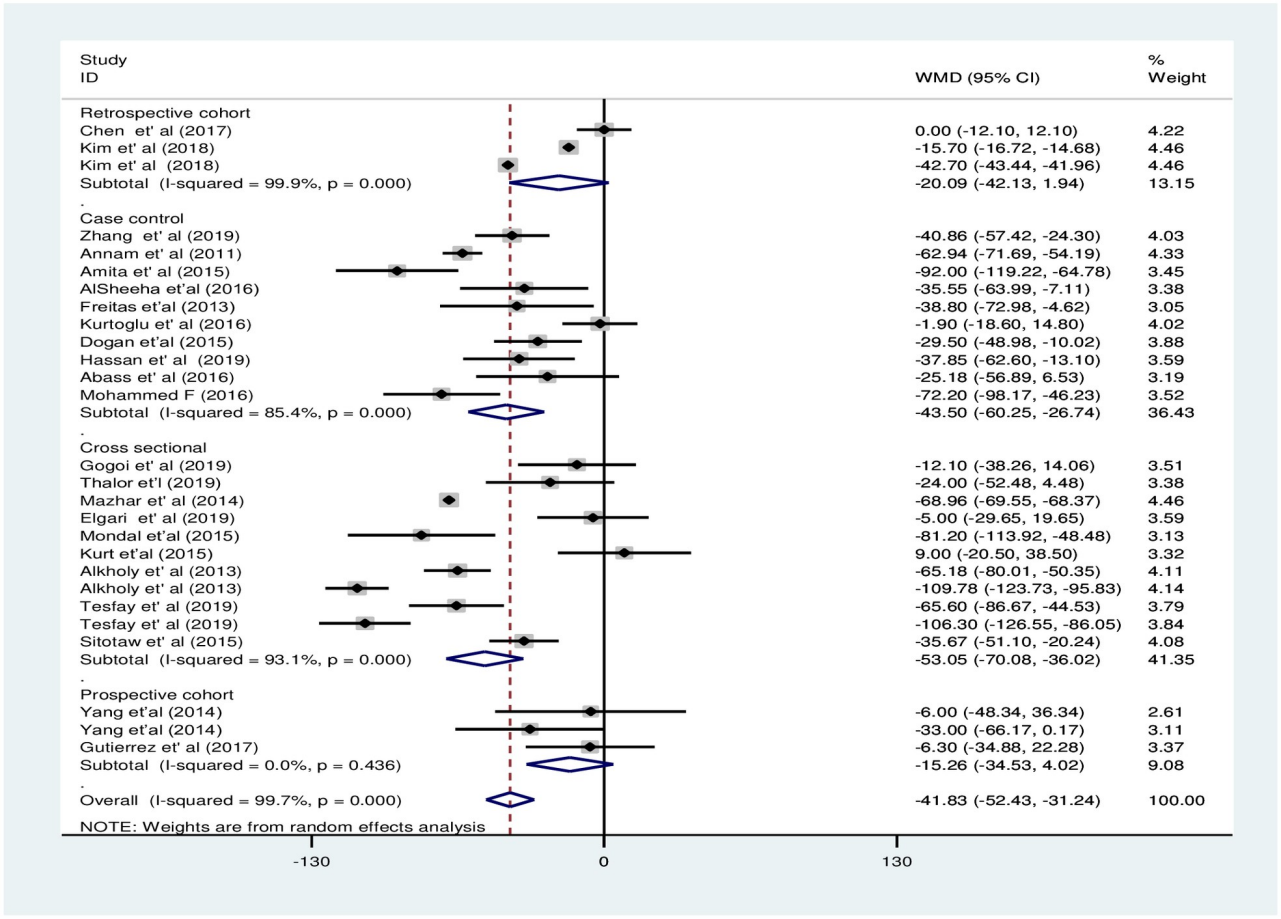
Forest plot of subgroup analysis for the pooled WMD estimate of PC by study design using random effect model.

In the subgroup analysis by PE severity, the pooled WMD was less evident among women with mild PE and NT pregnant women [WMD: -39.7×10^9^/L; 95%CI: -73.9,-5.4] compared to the difference between severe PE and NT pregnant women [WMD: -67.3×10^9^/L; 95%CI: -105.0, -29.5], the difference in both cases was statistically significant (Fig 6). The subgroup analysis by study continent showed that PC decreased significantly in PE groups in Asia and Africa with pooled WMD of -37.5×10^9^/L [95% CI; -52.2×10^9^/L, -22.9× 10^9^/L:I^2^ = 99.9%] and -65.6×10^9^/L [95% CI; -65.6×10^9^/L, -42.9×10^9^/L: I^2^ = 90.9%], respectively. However, the pooled WMD of PC was not significant in the Europe region [WMD: -8.8×10^9^/L; 95% CI;-30.7×10^9^/L, 12.9×10^9^/L] (Fig 7). Moreover, the subgroup analysis by study design showed that there was a significant decrement of PC in the PE group compared to the NT group in case-control and cross-sectional studies with a pooled WMD of -43.5×10^9^/L [95% CI; -60.3×10^9^/L, -26.7×10^9^/L] and -53.1×10^9^/L [95% CI; -70.1× 10^9^/L,-36.0× 10^9^/L], respectively. However, there was no significant difference among the groups in cohort studies with WMD -20.1×10^9^/L [95% CI; -42.1×10^9^/L, 1.9×10^9^/L] in the retrospective cohort and -15.3×10^9^/L [95% CI; -34.5× 10^9^/L, 4.0× 10^9^/L] in prospective cohort studies (Fig 8).

#### Subgroup analysis in estimated pooled weighted mean difference of mean platelet volume between preeclampsia and normotensive groups

**Fig 9:**
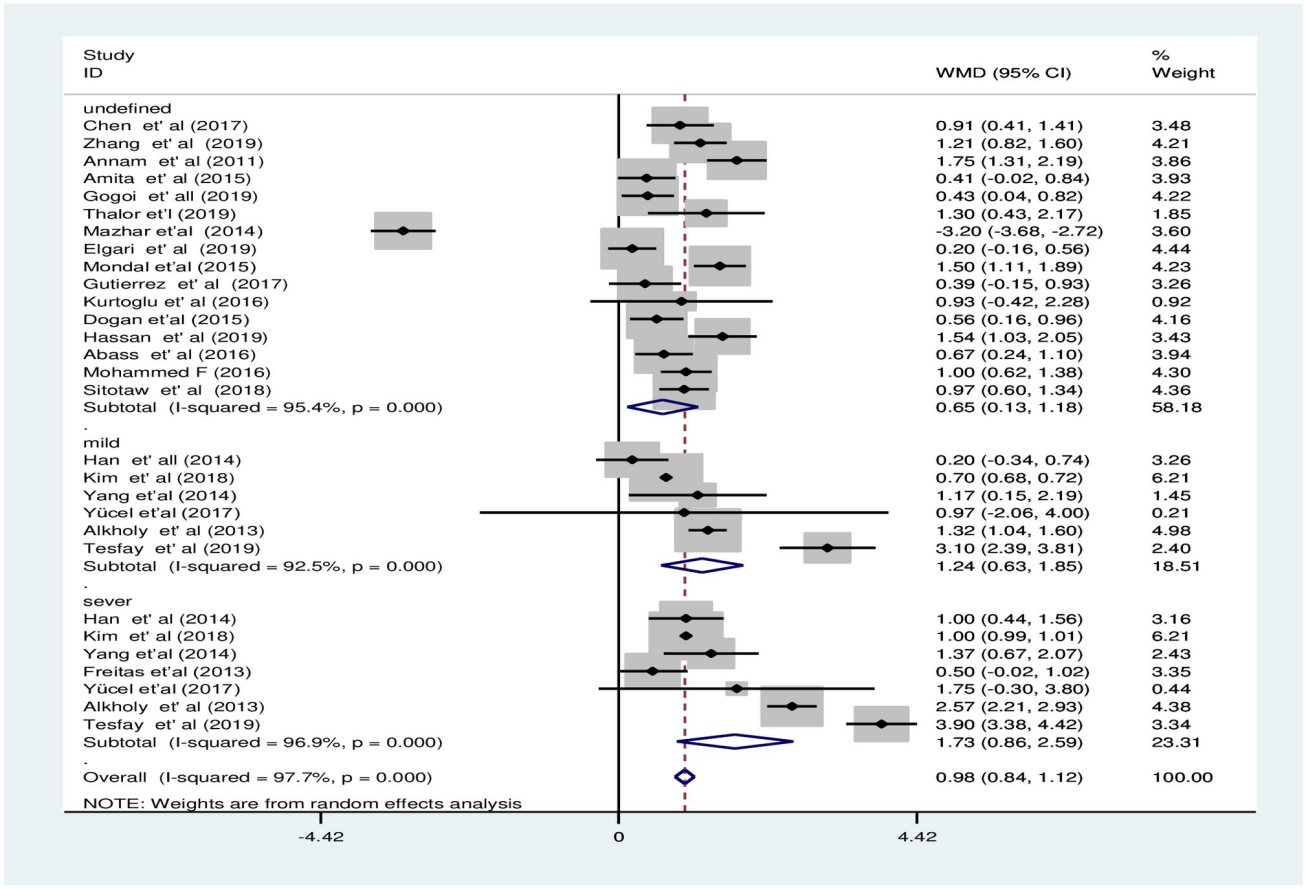
Forest plot of subgroup analysis for the pooled WMD estimate of MPV by PE severity using random effect model.

**Fig 10:**
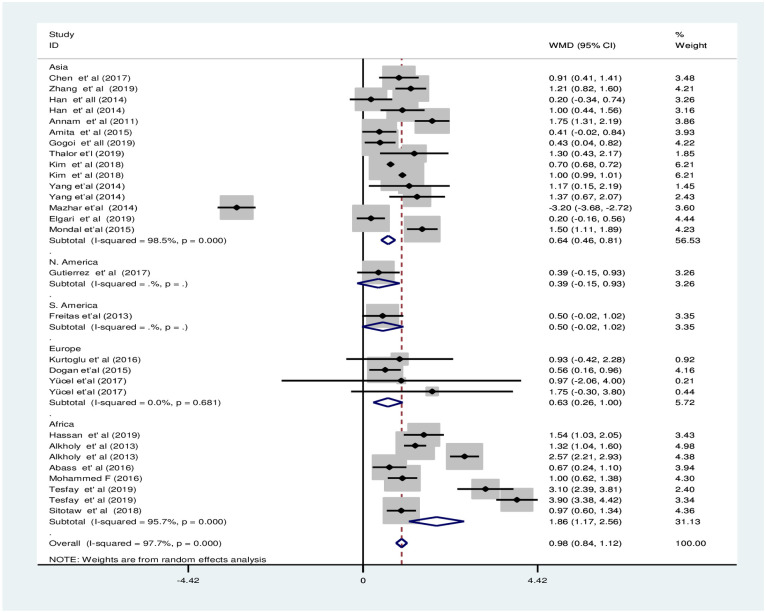
Forest plot of subgroup analysis for the pooled WMD estimate of MPV by continent using random effect model.

**Fig 11:**
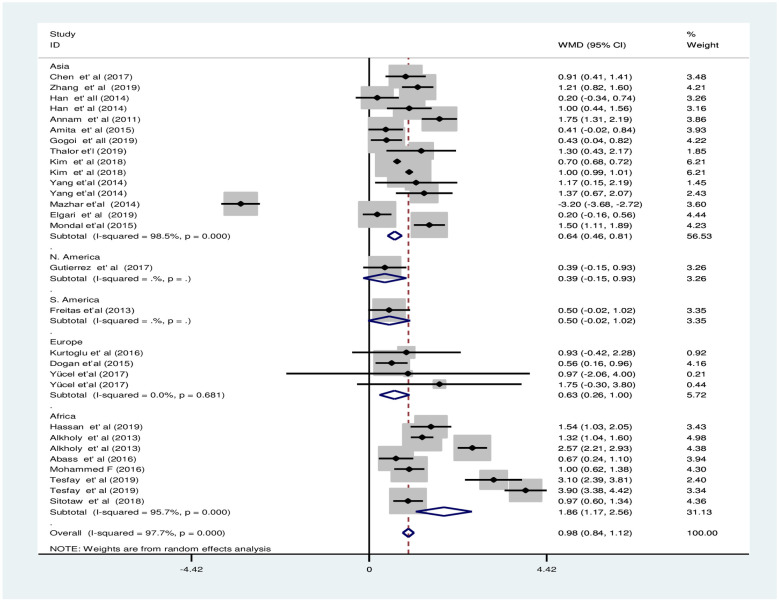
Forest plot of subgroup analysis for the pooled WMD estimate of MPV by study design using random effect model.

The subgroup analysis of MPV by PE severity revealed that the pooled WMD of MPV between severe PE group and NT group was smaller [WMD: 1.7fl; 95%CI; 0.9fl, 2.6fl] compared to the WMD between mild PE and NT groups [WMD: 1.2fl; 95%CI; 0.6fl, 1.8fl], the difference was significant in both subgroups (Fig 9). The subgroup analysis by continent showed that there was a significant difference of MPV between groups in Asia, Europe, and Africa with a pooled WMD of 0.6fl [95% CI; 0.5fl, 0.8fl], 0.6fl [95% CI; 0.3fl, 1fl], and 1.9fl [95% CI; 1.2fl, 2.6fl], respectively (Fig 10). Furthermore, the subgroup analysis by study design revealed that the pooled WMD among the groups in all study designs were significant with a pooled WMD of 0.9fl [95% CI; 0.6fl, 1.2fl], 1.2fl [95% CI; 0.1fl, 2.3fl], 0.9fl [95% CI; 0.6fl, 1.1fl], and 0.9fl [95% CI; 0.2fl, 1.6fl] in case-control, cross-sectional, retrospective cohort and prospective cohort studies, respectively (Fig 11).

### Sensitivity analysis

**Fig 12:**
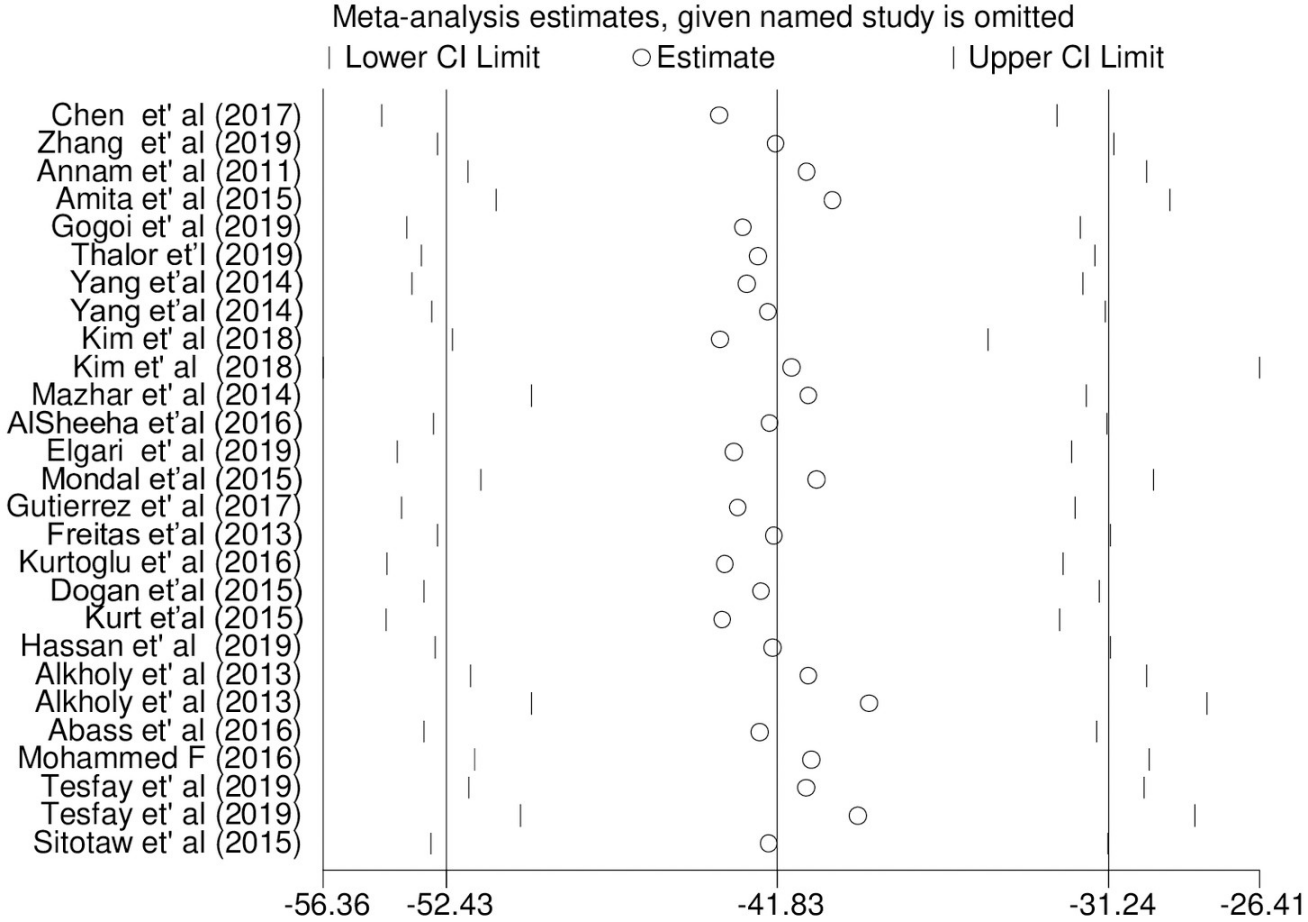
Sensitivity statistics of WMD analysis of PC between PE and NT groups.

**Fig 13:**
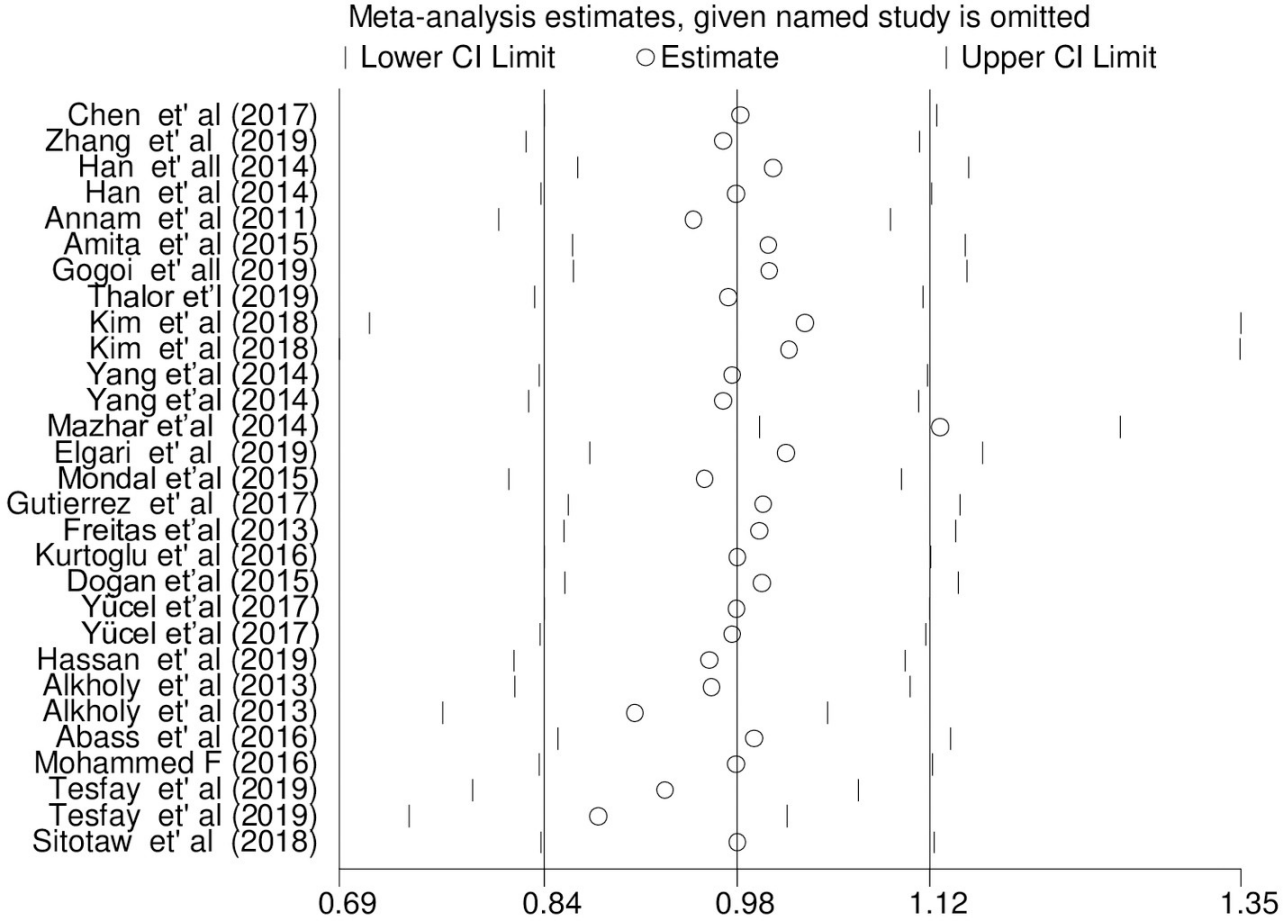
Sensitivity statistics of included studies for WMD analysis of MPV.

Sensitivity analysis was carried out on the pooled WMD of PC and MPV among PE and NT pregnant women. The analysis was done to evaluate the effect of each study on the estimated pooled WMD. The result showed that omitted studies did not show a significant effect on the pooled estimated WMD of PC analysis among PE and NT pregnant women (Fig 12). However, the sensitivity statistics on the pooled WMD of MPV analysis indicated that one study [70] has a determinant effect on the pooled estimated WMD of MPV with an estimate WMD of 1.13 which was out of the overall pooled estimated range [WMD: 0.98; 95% CI; 0.84, 1.124] (Fig 13).

## Discussions

During a normal pregnancy, the level of PC drops physiologically due to hemodilution, increased PLT consumption in peripheral tissue, and increased PLT aggregation as a result of increased levels of thromboxane A2 [71]. This Pregnancy-induced thrombocytopenia is mild and has no negative consequences for the mother or fetus, however, significant thrombocytopenia is associated with medical conditions and can have serious maternal-fetal consequences [72]. These changes may aggravate pregnancy-related disorders like PE. In established PE, changes in the coagulation system lead to a decrease in PC [24], which suggests an early sign of the disease [72]. Moreover, the progression of PE to the severe stage leads to increased turnover of PLTs [48]. The main objective of this systematic review and meta-analysis was to assess the association between PE and PC, PE and MPV. The association was determined by the pooled weighted mean difference. Accordingly, the majority of the included studies showed that PC was significantly lower in PE pregnant women than NT pregnant women [16, 17, 24, 25, 50, 57, 60, 61, 63, 65, 66, 68–70]. However, some studies showed that PC had not a significant difference between PE and NT pregnant women [26–28, 55, 56, 58, 62, 64]. Moreover, in the majority of the included studies, MPV was significantly higher in PE pregnant women than NT pregnant women [16, 17, 24, 25, 38, 50, 55, 56, 58, 60, 63–66, 68, 70]. Indeed, some studies showed MPV had no significant difference between PE and NT pregnant women [26, 28, 57, 59, 62, 69]. The reason for the discrepancy might be the difference in sample size, study design, diagnostic method, geographical location, and/or variation in gestational week between cases and controls.

Nevertheless, in this systematic review and meta-analysis, there was a significant decrement of pooled PC in the PE group compared to the NT group [WMD:-41.45 × 10^9^/L; 95% CI; -51.8, -31.0]. This could be due to increased consumption of PLT due to an abnormal coagulation system along with PLT activation [15, 18]. In preeclamptic women, plasma PLTs activation markers like β-thrombomodulin and PLT factor-4 significantly increased [69]. The increment of plasma PLTs activation markers like β-thrombomodulin and PLT factor-4 [73, 74]and the expression of activation markers on the PLTs surface confirm PLT activation [75, 76]. The activation of PLTs leads to PLT consumption. Besides, impaired endothelial synthesis of Prostacyclin and nitric oxide has been related to PE [69, 77]. Both Prostacyclin and nitric oxide relax blood vessels and inhibit PLT activation [78]. Therefore the impairment of the synthesis of these molecules in PE may cause blood vessel constriction and leads to PLT activation and consumption [15, 24].

Moreover, this systematic review and meta-analysis revealed that the overall pooled WMD of MPV values was significantly increased in the PE group when compared with the NT group [WMD: 0.98fl; 95%CI; 0.8, 1.1]. This demonstrated that PE is associated with a significant increase in MPV. The finding was in agreement with a systematic review and meta-analysis done by Bellos et al. which claimed that MPV was significantly higher in preeclamptic patients than the NT pregnant women [79]. This could be due to the increased PLT synthesis in the bone marrow and release of large PLTs as a result of increased PLT consumption and destruction, increasing MPV in PE [80].

In the subgroup analysis by PE severity, the pooled WMD of all PLT parameters was smaller among women with mild PE and NT pregnant women compared to the difference between severe PE and NT pregnant women. This might be due to the increased activation of the coagulation system as the disease progresses from mild to severe stage [1]. So, it makes the WMD of PLT parameters in severe PE higher and more significant. The subgroup analysis by study design showed that the pooled WMD of PC was not significant in both retrospective and prospective cohort studies while MPV was not significant only in retrospective cohort studies. Moreover, Subgroup analysis based on continent showed the pooled WMD of MPV was significant in all regions while the pooled WMD of PC between PE and NT groups was significant in Asia and Africa regions.

## Strengths and limitations of the study

The present review summarizes the current literature regarding the association of PC and MPV with PE which may give a clue to use these PLT parameters in the diagnosis of PE. It is based on a large number of studies for each parameter and included all relevant articles done around the globe. A comprehensive search using a different database and different searching strategies were the strength of this study. Moreover, the review was done in accordance with the protocol of the PRISMA statement. Nevertheless, most of the included studies had a case-control design; thus, selection bias cannot be excluded and studies that were published other than in English languages were not included. Even though subgroup analysis was performed the heterogeneity was still observed in all analyses.

## Conclusions

The pooled data from our systematic review and meta-analysis suggest that PE is associated with a lowering PC and increasing MPV. Thus, the measurement of PLT parameters, like PC and MPV, among pregnant women can be used as easily available, economical, and inexpensive clinical indicators in the assessment of PE. As a result, combining PLT Parameters with BP is more important than utilizing BP alone. However, the predictive performances like cut-off value, sensitivity, and specificity of these parameters have to be explored for the early diagnosis of PE. Further, multicenter longitudinal studies are required to evaluate their role at various GW of pregnancy.

## Supporting information

S1 File JBI critical appraisal tools. (DOCX)

S2 File The risk of bias assessment of included studies. (DOCX)

S1 Checklist PRISMA checklist 2020. (DOCX)

## Abbreviations

BP: Systolic Blood Pressure
CBC: Complete Blood Count
DBP: Diastolic blood pressure
GW: Gestational Week
IQR: Interquartile Range
JBI: Joanna Brigg Institute
Mesh: Medical Subject Headings
MPV: Mean Platelet Volume
NT: Normotensive
PC: Platelet Count
PE: Preeclampsia
PlGF: Placental Growth Factor
PLT: Platelet
PRISMA: Preferred Reporting Items for Systematic Review and Meta-Analysis
PROSPERO: International Prospective Register of Systematic Reviews
SBP: Blood Pressure
SD: Standard Deviation
SE: Standard Error
sEng: Soluble Endoglin
sFlt-1: Soluble fms-like Tyrosine Kinase 1
TGFβ: Transforming Growth Factor-β
VEGF: Vascular Endothelial Growth Factor
WMD: Weighted Mean Difference
