# Supplementary material for: Preeclampsia has an association with both platelet count and mean platelet volume: A systematic review and meta-analysis
Source: PLoS One. 2022 Sep 14;17(9):e0274398. doi: 10.1371/journal.pone.0274398 (PMC9473393; doi:10.1371/journal.pone.0274398)
Supplement: S2 File — (DOCX) [file pone.0274398.s002.docx]

## The risk of bias assessment results of the included cross sectional studies

| Author | Criteria for inclusion clearly defined? | Study subjects and setting clearly described? | The exposure measured in a valid and reliable way? | Standard criteria used for condition measurement? | Confounding factors identified? | Strategies to deal with confounding factors stated? | Outcomes measured validly and reliably? | Appropriate statistical analysis used? | Total score |
| --- | --- | --- | --- | --- | --- | --- | --- | --- | --- |
| Gogoi P et al (1) | 1 | 1 | 1 | 1 | 0 | 0 | 1 | 1 | 6/8 (75%) |
| Mazhar et al (2) | 1 | 1 | 1 | 1 | 0 | 0 | 1 | 1 | 6/8 (75%) |
| Elgari et al(3) | 1 | 1 | 1 | 1 | 0 | 0 | 1 | 1 | 6/8 (75%) |
| Mondalet’al(4) | 1 | 1 | 1 | 1 | 0 | 0 | 1 | 1 | 6/8 (75%) |
| Alkholy et al (5) | 1 | 1 | 1 | 1 | 0 | 0 | 1 | 1 | 6/8 (75%) |
| Kurt et’al(6) | 1 | 1 | 1 | 1 | 0 | 0 | 1 | 1 | 6/8 (75%) |
| Tesfay et' al (7) | 1 | 1 | 1 | 1 | 0 | 0 | 1 | 1 | 6/8 (75%) |
| Sitotawet' al (8) | 1 | 1 | 1 | 1 | 0 | 0 | 1 | 1 | 6/8 (75%) |

## The risk of bias assessment results of the included case-control studies

| Author | Groups were comparable? | Cases and control were matched? | The same criteria used to identify case and controls | Exposure measured in a standard? | Exposure measured in the same way? | Confounding factors identified? | Strategies to deal with confounding factors stated? | The outcome assessed in a standard way? | Exposure period long was enough | Appropriate statistical analysis used? | Total Score |
| --- | --- | --- | --- | --- | --- | --- | --- | --- | --- | --- | --- |
| Zhang et’l (9) | 1 | 1 | 1 | 1 | 1 | 0 | 0 | 1 | 1 | 1 | 8/10 (80%) |
| Annam et’l (10) | 1 | 0 | 1 | 1 | 1 | 0 | 0 | 1 | 1 | 1 | 7/10 (70%) |
| Amitaet’l (11) | 1 | 1 | 1 | 1 | 1 | 0 | 0 | 1 | 1 | 1 | 8/10 (80%) |
| Thaloret’l (12) | 1 | 1 | 1 | 1 | 1 | 0 | 0 | 1 | 1 | 1 | 8/10 (80%) |
| AlSheeha et’al(13) | 1 | 1 | 1 | 1 | 1 | 0 | 0 | 1 | 1 | 1 | 8/10 (80%) |
| Freitas et’al (14) | 1 | 1 | 1 | 1 | 1 | 0 | 0 | 1 | 1 | 1 | 8/10 (80%) |
| Kurtogluet’al (15) | 1 | 1 | 1 | 1 | 1 | 0 | 0 | 1 | 1 | 1 | 8/10 (80%) |
| Doganet’al (16) | 1 | 1 | 1 | 1 | 1 | 0 | 0 | 1 | 1 | 1 | 8/10 (80%) |
| Hassan et’l (17) | 1 | 0 | 1 | 1 | 1 | 0 | 0 | 1 | 1 | 1 | 7/10 (70%) |
| Abass et' al (18) | 1 | 1 | 1 | 1 | 1 | 0 | 0 | 1 | 1 | 1 | 8/10 (80%) |
| Mohammed F (19) | 1 | 0 | 1 | 1 | 1 | 0 | 0 | 1 | 1 | 1 | 7/10 (70%) |
| Han et al (20) | 1 | 1 | 1 | 1 | 1 | 0 | 0 | 1 | 1 | 1 | 8/10 (80%) |

## The risk of bias assessment results of the included Cohort studies

| Author | Groups recruited from the same population? | Did exposure measure similarly to assign people to both groups? | Exposure measured in a valid and reliable way? | Confounding factors identified? | Strategies to deal with confounding factors stated? | Participants were free of the outcome at start of study? | Outcome measured in a valid and reliable way? | Follow up time was reported and sufficient | Was follow up complete, and if not, reasons described | Strategies to address incomplete follow up utilized | appropriate statistical analysis used | Total score |
| --- | --- | --- | --- | --- | --- | --- | --- | --- | --- | --- | --- | --- |
| Chen et’l (21) | 1 | 1 | 1 | 0 | 0 | 1 | 1 | 1 | 1 | 0 | 1 | 8/11 (72.3) |
| Kim et’al (22) | 1 | 1 | 1 | 0 | 0 | 1 | 1 | 1 | 1 | 0 | 1 | 8/11 (72.3) |
| Yang et’al (23) | 1 | 1 | 1 | 0 | 1 | 1 | 1 | 1 | 1 | 0 | 1 | 9/11 (81.8) |
| Gutierrez et al (24) | 1 | 1 | 1 | 0 | 0 | 1 | 1 | 1 | 1 | 0 | 1 | 8/11 (72.3) |
| Yücelet’al (25) | 1 | 1 | 1 | 0 | 0 | 1 | 1 | 1 | 1 | 0 | 1 | 8/11 (72.3) |

1. Gogoi P, Sinha P, Gupta B, Firmal P, Rajaram S. Neutrophil‐to‐lymphocyte ratio and platelet indices in pre‐eclampsia. International Journal of Gynecology Obstetrics. 2019;144(1):16-20.

2. Mazhar S, Mazhar N, Anwa A. Usefulness of platelet count and mean platelet volume in prediction of preeclampsia. Pakistan Postgraduate Medical Journal. 2014;25(4):118-20.

3. Elgari M, Khabour O, Alhag S. Correlations between changes in hematological indices of mothers with preeclampsia and umbilical cord blood of newborns. Clinical Experimental Hypertension. 2019;41(1):58-61.

4. Mondal B, Paul D, Sultana T, Rahman Q, Ahmed S, Fatema K, et al. Assessment of platelet count and platelet indices in preeclampsia and eclampsia. Am J Innov Res Appl Sci 2015;1(3):80-4.

5. Alkholy EA-M, Farag E, Behery M, Ibrahim M. The significance of platelet count, mean platelet volume and platelet width distribution in Preeclampsia. AAMJ. 2013;11(1).

6. Kurt R, Aras Z, Silfeler D, Kunt C, Islimye M, Kosar O. Relationship of red cell distribution width with the presence and severity of preeclampsia. Clinical Applied Thrombosis/Hemostasis. 2015;21(2):128-31.

7. Tesfay F, Negash M, Alemu J, Yahya M, Teklu G, Yibrah M, et al. Role of platelet parameters in early detection and prediction of severity of preeclampsia: A comparative cross-sectional study at Ayder comprehensive specialized and Mekelle general hospitals, Mekelle, Tigray, Ethiopia. Plos one. 2019;14(11):236.

8. Sitotaw C, Asrie F, Melku M. Evaluation of platelet and white cell parameters among pregnant women with Preeclampsia in Gondar, Northwest Ethiopia: A comparative cross-sectional study. Pregnancy hypertension. 2018;13:242-7.

9. Zhang H, Zhang Y, Wang Z, Yan J. Platelet count and mean platelet volume predict atypical pre-eclampsia. Pregnancy hypertension. 2019;18:29-34.

10. Annam V, Srinivasa K, Yatnatti S, Med S. Evaluation of platelet indices and platelet counts and their significance in pre-eclampsia and eclampsia. Int J Biol Med Res. 2011;2(1):425-8.

11. Amita K, Kumar N, Shobha S, Shankar V. The role of platelet parameters as a biomarker in the diagnosis and in predicting the severity of preeclampsia. Indian Journal of Pathology Oncology. 2015;2(2):57-60.

12. Thalor N, Singh K, Pujani M, Chauhan V, Agarwal C, Ahuja R. A correlation between platelet indices and preeclampsia. Hematology, transfusion cell therapy. 2019;41(2):129-33.

13. AlSheeha M, Alaboudi R, Alghasham M, Iqbal J, Adam I. Platelet count and platelet indices in women with preeclampsia. Vascular health risk management. 2016;12:477.

14. Freitas L, Alpoim P, Komatsuzaki F, Carvalho M, Dusse LM. Preeclampsia: are platelet count and indices useful for its prognostic? Hematology. 2013;18(6):360-4.

15. Kurtoglu E, Kokcu A, Celik H, Bildircin F, Tosun M, Alper T, et al. Validity of platelet indices in predicting the risk of developing preeclampsia. Clinical Research. 2016;33(2):57-61.

16. Doğan K, Guraslan H, Senturk M, Helvacioglu C, İdil S, Ekin M. Can platelet count and platelet indices predict the risk and the prognosis of preeclampsia? Hypertension in pregnancy. 2015;34(4):434-42.

17. Hassan HE-S, Azzam H, Othman M, Hassan M, Selim T. Soluble E-selectin, platelet count and mean platelet volume as biomarkers for pre-eclampsia. Pregnancy hypertension. 2019;17:1-4.

18. Abass A-E, Abdalla R, Omer I, Ahmed S, Khalid A, Elzein H. Evaluation of platelets count and indices in pre-eclampsia compared to normal pregnancies. IOSR J Dent Med Sci. 2016;1(15):5-8.

19. Mohammed F. Platelets Count and Indices as possible predictors for pre-eclampsia in Sudanese Women in Khartoum State Maternity Hospitals: Sudan University of Science & Technology; 2016.

20. Han L, Liu X, Li H, Zou J, Yang Z, Han J, et al. Blood coagulation parameters and platelet indices: changes in normal and preeclamptic pregnancies and predictive values for preeclampsia. PloS one. 2014;9(12):e114488.

21. Chen Y, Lin L. Potential value of coagulation parameters for suggesting preeclampsia during the third trimester of pregnancy. The American journal of the medical sciences. 2017;354(1):39-43.

22. Kim MA, Han GH, Kwon JY, Kim YH. Clinical significance of platelet‐to‐lymphocyte ratio in women with preeclampsia. American Journal of Reproductive Immunology. 2018;80(1):e12973.

23. Yang S, Cho S, Kwon H, Sohn I, Hwang H. Significance of the platelet distribution width as a severity marker for the development of preeclampsia. European Journal of Obstetrics Gynecology Reproductive Biology. 2014;175:107-11.

24. Gutierrez-Aguirre C, García-Lozano J, Treviño-Montemayor O, Iglesias-Benavides J, Cantú-Rodríguez O, González-Llano O, et al. Comparative analysis of iron status and other hematological parameters in preeclampsia. Hematology. 2017;22(1):36-40.

25. Yücel B, Ustun B. Neutrophil to lymphocyte ratio, platelet to lymphocyte ratio, mean platelet volume, red cell distribution width and plateletcrit in preeclampsia. Pregnancy Hypertension: An International Journal of Women's Cardiovascular Health. 2017;7:29-32.
